# Planning-Based Dosimetric Comparison of Three-Dimensional Conformal Radiotherapy (Flattened Versus Unflattened Beams), Intensity-Modulated Radiotherapy, and Volumetric Modulated Arc Therapy for Pituitary Adenomas on Halcyon

**DOI:** 10.7759/cureus.109398

**Published:** 2026-05-21

**Authors:** Deepika Malik, Naveen V Prasath, Mahendran Chinnamuthu, Rishabh Mahajan, Gomathi Rajendiran, Priyusha Bagdare

**Affiliations:** 1 Radiation Oncology, Sri Aurobindo Medical College and Post-graduate Institute, Indore, IND; 2 Medical Physics, Sri Aurobindo Medical College and Post-graduate Institute, Indore, IND

**Keywords:** electronically flattened beam, intensity-modulated radiotherapy, multileaf collimators, pituitary neoplasms, volumetric modulated arc therapy

## Abstract

This study was undertaken to perform a dosimetric comparison of four distinct radiotherapy planning strategies for pituitary adenomas on the Varian Halcyon platform (Varian Medical Systems, Palo Alto, CA, USA): three-dimensional conformal radiotherapy (3DCRT) using electronically flattened beams, 3DCRT using true flattening filter-free (FFF) beams, intensity-modulated radiotherapy (IMRT), and volumetric modulated arc therapy (VMAT). The study additionally evaluated the feasibility and dosimetric implications of reproducing conventional forward-planned beam characteristics on an inherently FFF platform using dynamic multileaf collimator (MLC)-based electronic fluence shaping. Planning data from 15 patients were utilized. Four plans were generated per patient: 3DCRT with electronically flattened beams, 3DCRT with true FFF beams, IMRT, and VMAT. A prescription dose of 54 Gy in 30 fractions was applied with ≥95% planning target volume (PTV) coverage. Plan evaluation included D_2%_, D_50%_, D_98%_, conformity index (CI), homogeneity index (HI), gradient index (GI), and doses to optic nerves, chiasm, lenses, cochleae, brain, and brainstem. All techniques achieved clinically acceptable coverage (V_95%_ > 99%). IMRT and VMAT showed superior conformity compared with 3DCRT (p < 0.0001), with VMAT achieving the lowest HI. IMRT provided the lowest mean doses to lenses. VMAT achieved the lowest maximum doses to optic nerves, eyes, brain, and brainstem, and the lowest mean doses to the chiasm and brainstem. True 3DCRT FFF required fewer monitor units (MUs) and had sharper gradients, whereas electronically flattened plans were more uniform. Inverse-planned techniques provide superior organ-at-risk (OAR) sparing in pituitary adenomas. VMAT offers superior normal tissue dose constraints. Their complementary strengths suggest that technique selection should be guided by anatomical considerations and proximity of critical structures, emphasizing individualized planning rather than reliance on a single modality. Significant dosimetric differences exist between electronically flattened and true 3DCRT FFF delivery, underscoring the importance of beam model selection in forward-planned radiotherapy for pituitary adenomas.

## Introduction

Pituitary adenomas are benign tumors arising from the anterior pituitary gland and constitute approximately 10%-15% of all intracranial neoplasms [1]. Although often non-invasive and slow-growing, these tumors may cause significant clinical symptoms due to hormone hypersecretion or compression of adjacent critical structures, such as the optic chiasm and hypothalamus. Surgical resection remains the primary treatment for symptomatic and hormonally active adenomas; however, complete excision is not always feasible due to the proximity of vital neurovascular structures. In such cases, adjuvant radiotherapy plays a pivotal role in controlling residual or recurrent disease [2].

Over the past two decades, radiation therapy techniques have evolved considerably, allowing for more conformal dose distributions and better sparing of surrounding normal tissues. Three-dimensional conformal radiotherapy (3DCRT) has been widely used due to its simplicity and accessibility, but it often delivers higher doses to nearby critical organs compared to more advanced techniques. Intensity-modulated radiotherapy (IMRT) improves dose conformity and homogeneity by modulating beam intensities, thereby allowing better sparing of organs at risk (OARs) such as the optic nerves, chiasm, and brainstem [3]. More recently, volumetric modulated arc therapy (VMAT), including RapidArc, has been introduced, enabling continuous delivery of radiation as the gantry rotates around the patient, further enhancing dose conformity and reducing treatment time [4].

There is increasing adoption of VMAT techniques such as RapidArc in clinical practice, and we intended to compare the relative planning-based dosimetric benefits of RapidArc compared to IMRT on Halcyon (Varian Medical Systems, Palo Alto, CA, USA). Although previous studies have demonstrated improved conformity and OAR sparing with IMRT and VMAT compared with conventional 3DCRT in pituitary adenomas, limited data exist regarding the dosimetric implications of electronically generated beam flattening on inherently flattening filter-free (FFF) linear accelerator platforms such as the Varian Halcyon. Unlike conventional linear accelerators that rely on a physical flattening filter, the Halcyon system employs dynamic multileaf collimator (MLC)-based fluence modulation to generate electronically flattened beam profiles. The extent to which such electronically flattened delivery can reproduce conventional forward-planning dosimetric characteristics, while balancing dose homogeneity, conformity, gradient falloff, OAR sparing, and delivery efficiency, remains insufficiently characterized.

Accordingly, this study aimed to perform a comprehensive dosimetric comparison of four distinct radiotherapy planning strategies for pituitary adenomas on the Halcyon platform: 3DCRT using electronically flattened beams, 3DCRT using true FFF beams, IMRT, and VMAT. In addition to comparing target coverage and OAR sparing, the study specifically evaluated the feasibility and dosimetric consequences of generating conventional forward-planned dose profiles through dynamic MLC-driven electronic beam flattening. A detailed dosimetric analysis will help clarify the potential advantages of advanced techniques and guide optimal treatment planning strategies for patients with pituitary adenomas.

## Materials and methods

This study was conducted using planning data from 15 pituitary adenoma patients previously treated with radiotherapy at our center. As it was a retrospective study based on planning data and not the actual patients, ethical committee approval was not sought. All such patients had undergone high-resolution contrast-enhanced computed tomography (CT) simulation scans, fused with contrast-enhanced magnetic resonance imaging (MRI) scans for accurate target delineation. Inclusion criteria included histologically confirmed pituitary adenoma, postoperative or planned in a multidisciplinary tumor board for non-surgical management, and availability of complete imaging and planning datasets.

Target and OAR delineation

The gross tumor volume (GTV) was defined as the visible tumor on fused MRI-CT images. The clinical target volume (CTV) included the GTV with a margin accounting for potential microscopic spread, typically 2-3 mm, and the planning target volume (PTV) was generated by adding an additional isotropic margin of 2-3 mm around the CTV. For operated patients, the CTV included the GTV (if residual) and the tumor bed, and it was then expanded symmetrically by 2-3 mm to create the PTV to account for setup errors. OARs contoured included the bilateral eyes, bilateral lens, bilateral optic nerves, optic chiasm, bilateral cochlea, brainstem, and normal brain tissue.

Treatment Planning Techniques

Distinct radiotherapy treatment plans were developed using the Eclipse Treatment Planning System (TPS; Version 17.0.1, Varian Medical Systems): 3DCRT with flattened beams, 3DCRT with unflattened beams, IMRT, and VMAT. To avoid any planner bias, all plans were generated by a single physicist. Each patient was prescribed a total radiation dose of 54 Gy, delivered over 30 fractions, using the Varian Halcyon Elite linear accelerator. All plans were optimized to ensure that the PTV received at least 95% of the prescribed dose. Dose constraints for critical organs were maintained as follows: maximum doses to the brain, brainstem, bilateral optic nerves, optic chiasm, and both eye lenses were limited to 60, 54, 54, 55, and 7 Gy, respectively. Additionally, the mean dose to both cochleae and the orbits of the eyes was restricted to not exceed 45 and 35 Gy, respectively.

Unlike conventional linear accelerators that employ a physical flattening filter, the Varian Halcyon platform delivers an inherently FFF photon beam and generates a clinically flattened fluence profile through predefined dynamic MLC modulation, effectively introducing fluence shaping at the level of beam delivery rather than within the treatment head. Fluence shaping was achieved through the inverse planning Photon Optimization (PO; v17.0.1) algorithm and beam profile adjustment using Eclipse beam modeling, and the methodology was based on the TPS-driven fluence modulation PO (v17.0.1) algorithm. All 3DCRT plans were designed using a four-field technique, employing gantry angles of 0°, 90°, 180°, and 270°, with both 6 MV flattened (FF) and FFF photon beams. The collimator angle was consistently maintained at 0° for each gantry position. To reduce unnecessary radiation exposure to healthy brain tissue, the weight of the posterior-anterior (PA) field was adjusted accordingly.

For the IMRT plans, five fields were employed with gantry angles of 0°, 60°, 150°, 210°, and 300°. During the optimization process, entry and exit doses through the eye lenses were specifically controlled to minimize the maximum dose to these structures. This strategy also contributed to lowering the doses to the optic nerves and eye orbits.

**Figure 1:**
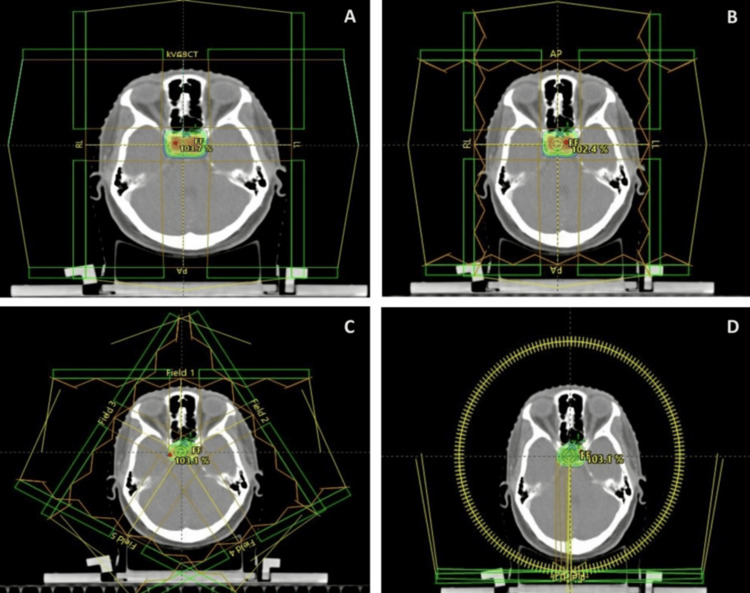
Field arrangements and dose wash for (A) three-dimensional conformal radiotherapy (3DCRT) using electronically flattened beams, (B) 3DCRT using true flattening filter-free (FFF) beams, (C) intensity-modulated radiotherapy (IMRT), and (D) volumetric modulated arc therapy (VMAT) plans.

VMAT plans were generated using a dual-arc technique with 6 MV FFF photon beams. Each plan consisted of two arcs: one rotating clockwise (CW) from 181° to 179° and the other rotating counterclockwise (CCW) from 179° to 181°. To prevent radiation leakage from the MLC interleaves from concentrating on a single plane during gantry rotation, the collimator angles were set to 30° for the CW arc and 330° for the CCW arc. For the VMAT plans, beam-hold avoidance sectors were also used for enhanced eye lens sparing. Separate VMAT plans incorporating a ±15 to 25° avoidance sector were generated for bilateral lens protection. For the CW arcs, avoidance sectors of 315°-340° and 20°-45° were applied to minimize radiation exposure to the right and left eye lenses, respectively. Similarly, for the CCW arcs, avoidance sectors of 45°-20° and 340°-325° were employed. All four treatment techniques were planned and delivered using a dose rate of 800 monitor units (MUs)/min. A schematic representation of the beam configurations adopted for all planning techniques is illustrated in Figure 1.

Plan evaluation and dosimetric comparison

Dose-volume histograms (DVHs) were generated for all treatment plans using the DVH estimation algorithm, Version 17.0.1, to assess both target volumes and OARs. Various dosimetric parameters-including the homogeneity index (HI), conformity index (CI), and gradient index (GI)-were derived from the DVH data. For OAR evaluation, the maximum and mean doses delivered to the brain, brainstem, bilateral eye lenses, bilateral orbits of the eyes, bilateral optic nerves, bilateral cochlea, and optic chiasm were analyzed.

Homogeneity Index

The HI [5], calculated using the method recommended by International Commission on Radiation Units and Measurements (ICRU) Report 83, was used to evaluate the consistency of dose distribution within the target volume. According to this guideline, the ideal HI value is 0, and it is calculated as

$HI = \frac{D_{2\%} - D_{98\%}}{D_{50\%}}$

where D_2%_ = dose received by 2% of the PTV (indicating the near-maximum dose), D_98%_ = dose received by 98% of the PTV (indicating the near-minimum dose), and D_50%_ = dose received by 50% of the PTV.

Conformity Index

The CI assesses how precisely the prescribed radiation dose conforms to the shape of the target volume. This study utilized the formula introduced by Ian Paddick [6], which suggests an ideal CI value of 1:

$CI = \frac{(TV_{\mathrm{PIV}})^2}{TV \times PIV}$

where TV_PIV_ = volume of the prescribed isodose that overlaps with the target volume, TV = total target volume (PTV), and PIV = volume enclosed by the prescribed isodose.

Gradient Index

The GI quantifies how rapidly the dose falls off outside the target, reflecting the sharpness of the dose gradient. Also based on Paddick and Lippitz’s formula [7], a GI value below 3 is generally considered acceptable:

$GI = \frac{V_{50\%}}{PIV}$

where V_50%_ = volume covered by 50% of the prescription dose and PIV = volume enclosed by the full prescription dose.

Integral Dose

The integral dose (ID) to each OAR was calculated as the product of the mean dose and organ volume, a widely accepted method for estimating total energy deposition in normal tissues [5]:

$ID = D \times V$

where D̄ = mean dose and V = volume of the particular OAR.

Statistical analysis

The acquired data were expressed in the form of mean ± standard deviation and analyzed in Minitab Statistical Software Version 22.1.0 (Minitab, LLC, State College, PA, USA). Initially, data for each group were assessed for normality using the Shapiro-Wilk test. If data were normally distributed, the assumption of homogeneity of variance was evaluated using Levene’s test. When both assumptions of normality and equal variance were met, one-way ANOVA was employed to compare the means across the four radiotherapy techniques. If the normality assumption was violated, the non-parametric Kruskal-Wallis test was used. Additionally, when normality was satisfied but equal variance was not, Welch’s ANOVA was performed to account for unequal variances. This structured approach ensured the application of appropriate statistical tests based on the distributional properties and variance behavior of data. A p-value less than 0.05 was considered indicative of a statistically significant difference between the groups.

## Results

All evaluated planning techniques-3DCRT using electronically flattened beams, 3DCRT using true FFF beams, IMRT, and VMAT-achieved clinically acceptable treatment plans, with adequate PTV coverage in all cases (V_95%_ > 99%). No statistically significant differences were observed in maximum PTV dose or D_98%_, confirming consistent target dose coverage across all modalities.

Significant variations were observed in several PTV dose metrics. The minimum, mean, D_2%_, and D_50%_ doses differed significantly among techniques (p < 0.01), with 3DCRT FFF generally exhibiting higher central dose values, while IMRT demonstrated comparatively lower minimum PTV doses. Dose conformity was significantly improved with inverse-planned techniques, with VMAT and IMRT achieving higher CI values compared with both 3DCRT approaches (p < 0.0001). Dose homogeneity also varied significantly, with VMAT demonstrating the most homogeneous dose distribution and 3DCRT FFF exhibiting the highest heterogeneity. GI values were significantly lower for 3DCRT FFF, indicating steeper dose falloff, although all techniques achieved clinically acceptable gradient indices.

MU requirements differed significantly between techniques (p < 0.0001). 3DCRT FFF required the fewest MUs, followed by 3DCRT with electronically flattened beams, whereas IMRT required the highest MU, reflecting differences in delivery efficiency (Table 1).

**Table 1:** Comparison of the four techniques for PTV coverage. Radiation doses in Gray, represented as mean ± SD; mean ± SD are compared for target volume coverage between different planning techniques. A p-value of <0.05 was considered significant. SD: standard deviation; PTV: planning treatment volume; 3DCRT FFF: three-dimensional conformal radiotherapy using flattening filter-free beams; 3DCRT FF: three-dimensional conformal radiotherapy using flattened beams; VMAT: volumetric modulated arc therapy; IMRT: intensity-modulated radiotherapy; D_2%_, D_50%_, and D_98%_: dose received by 2%, 50%, and 98% volume of PTV; V_95%_, V_107%_: volume of the PTV receiving 95% and 107% of the prescribed dose; CI: conformity index; HI: homogeneity index; GI: gradient index; H: test statistic numerical value for Kruskal-Wallis test; F: test statistic numerical value for ANOVA test

PTV parameters	3DCRT FFF (mean ± SD)	3DCRT FF (mean ± SD)	VMAT (mean ± SD)	IMRT (mean ± SD)	p-value	Test statistic numerical value
Minimum (cGy)	4,994.14 ± 225.42	4,924.52 ± 266.74	4,614.57 ± 385.68	4,455.42 ± 507.3	<0.0001	H = 24.5357
Mean (cGy)	5,510.59 ± 67.76	5,450.53 ± 63.22	5,469.77 ± 28.06	5,452.64 ± 14.61	0.0031	F = 6.4653
Maximum (cGy)	5,689.51 ± 86.92	5,640.35 ± 68.41	5,683.53 ± 79.43	5,698.51 ± 52.27	0.0652	F = 2.508
D_2%_ (cGy)	5,662.89 ± 71.68	5,582.48 ± 69.47	5,580.36 ± 27.61	5,588.92 ± 40.50	0.0004	F = 10.1712
D_50%_ (cGy)	5,523.16 ± 61.78	5,461.76 ± 70.35	5,476.11 ± 20.81	5,468.31 ± 33.14	0.0025	F = 6.9902
D_98%_ (cGy)	5,228.17 ± 58.83	5,251.74 ± 48.83	5,259.89 ± 51.35	5,225.07 ± 85.26	0.2194	F = 1.5078
V_95%_ (%)	99.45 ± 0.77	99.72 ± 0.33	99.50 ± 0.48	99.03 ± 0.90	0.0149	H = 10.4781
CI	0.57 ± 0.09	0.54 ± 0.06	0.79 ± 0.09	0.77 ± 0.06	<0.0001	F = 57.8136
HI	0.08 ± 0.01	0.06 ± 0.01	0.06 ± 0.01	0.07 ± 0.02	<0.0001	F = 9.1172
GI	0.57 ± 0.10	0.54 ± 0.07	0.80 ± 0.09	0.79 ± 0.07	<0.0001	F = 58.569
Monitor units (MUs)	261.73 ± 7.82	316.09 ± 10.92	495.11 ± 39.27	733.25 ± 144.46	<0.0001	F = 160.1042

**Figure 2:**
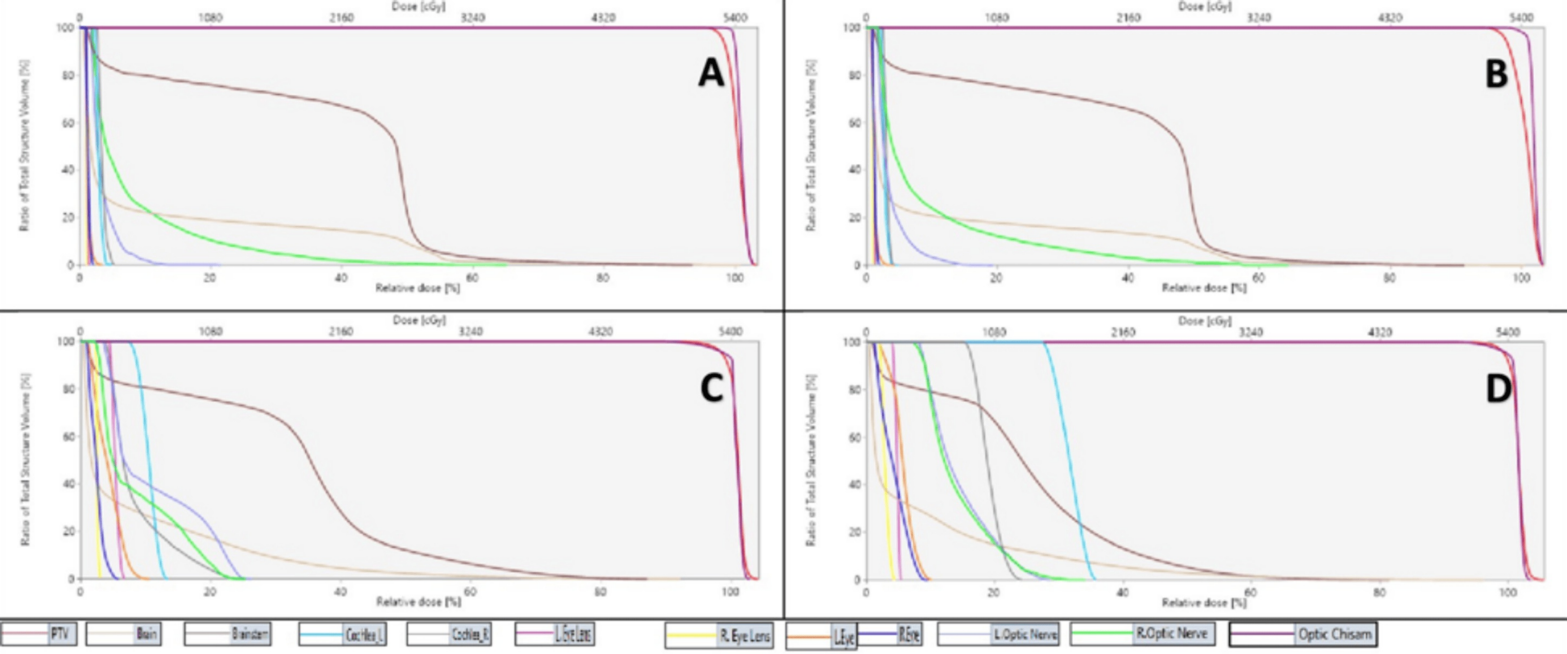
Dose-volume histogram for (A) three-dimensional conformal radiotherapy (3DCRT) using electronically flattened beams, (B) 3DCRT using true flattening filter-free (FFF) beams, (C) intensity-modulated radiotherapy (IMRT), and (D) volumetric modulated arc therapy (VMAT) plans.

All treatment techniques produced clinically acceptable plans while satisfying OAR constraints. Significant dose differences were observed across most structures. IMRT achieved the lowest maximum and mean doses to both eye lenses, indicating superior sparing of small radiosensitive structures. VMAT provided the lowest maximum doses to both optic nerves, eye globes, brain, and brainstem, and also achieved the lowest mean dose to the right optic nerve, optic chiasm, and brainstem. For cochleae, 3DCRT FFF consistently demonstrated the lowest maximum and mean doses bilaterally. Mean brain dose was lowest with IMRT, although this difference was not statistically significant. Overall, inverse-planned techniques showed improved organ sparing compared with 3D-conformal approaches, with IMRT favoring small critical structures and VMAT providing better control of maximum doses to larger intracranial organs, while 3DCRT FFF offered superior cochlear sparing (Table 2). The DVH for the four techniques is represented in Figure 2.

**Table 2:** Comparison of the four techniques for OAR constraints. Radiation doses in Gray, represented as mean ± SD; mean ± SD are compared for various OARs between different planning techniques. p-value less than 0.05 is considered significant. 3DCRT FFF: three-dimensional conformal radiotherapy using flattening filter-free beams; 3DCRT FF: three-dimensional conformal radiotherapy using flattened beams; VMAT: volumetric modulated arc therapy; IMRT: intensity-modulated radiotherapy; OAR: organ at risk; SD: standard deviation; H: test statistic numerical value for Kruskal-Wallis test; F: test statistic numerical value for ANOVA test

OARs	Parameters	3DCRT FFF (mean ± SD)	3DCRT FF (mean ± SD)	VMAT (mean ± SD)	IMRT (mean ± SD)	p-value	Test statistic numerical value
Left eye lens	Max dose (Gy)	1.89 ± 1.08	2.02 ± 1.06	2.92 ± 0.84	1.77 ± 0.78	0.0009	H = 16.315
	Mean dose (Gy)	1.20 ± 0.46	1.36 ± 0.51	2.23 ± 0.73	1.32 ± 0.52	<0.0001	F = 14.1478
Right eye lens	Max dose (Gy)	2.07 ± 1.65	2.09 ± 1.44	2.79 ± 0.73	1.62 ± 0.72	0.0030	H = 13.9269
	Mean dose (Gy)	1.28 ± 0.66	1.41 ± 0.65	2.17 ± 0.64	1.24 ± 0.39	<0.0001	F = 10.6808
Left optic nerve	Max dose (Gy)	39.87 ± 16.02	39.53 ± 15.89	22.89 ± 6.31	24.10 ± 7.39	0.0001	H = 22.398
	Mean dose (Gy)	13.74 ± 8.72	14.24 ± 10.10	12.07 ± 3.63	11.79 ± 4.57	0.0005	F = 0.555
	Integral dose (Gy cm^3^)	19.75 ± 12.20	19.65 ± 12.23	11.70 ± 7.08	12.00 ± 6.88	0.0089	F = 4.152
Right optic nerve	Max dose (Gy)	42.20 ± 16.23	41.30 ± 15.88	25.14 ± 10.34	26.56 ± 10.85	0.0001	H = 21.271
	Mean dose (Gy)	16.58 ± 13.37	17.75 ± 15.15	13.83 ± 6.26	14.59 ± 7.57	0.9855	H = 0.1478
	Integral dose (Gy cm^3^)	26.18 ± 19.52	25.68 ± 19.51	16.05 ± 13.96	16.56 ± 13.82	0.0996	F = 2.1605
Left cochlea	Max dose (Gy)	8.02 ± 9.35	8.91 ± 9.72	12.92 ± 6.76	11.73 ± 5.52	0.0061	H = 12.385
	Mean dose (Gy)	4.39 ± 6.18	4.99 ± 6.42	10.43 ± 6.37	9.11 ± 5.28	<0.0001	H = 35.078
	Integral dose (Gy cm^3^)	0.90 ± 1.37	0.99 ± 1.48	1.37 ± 1.38	1.09 ± 1.02	0.7070	F = 4.6858
Right cochlea	Max dose (Gy)	10.28 ± 12.06	10.95 ± 12.59	14.07 ± 7.81	12.81 ± 6.42	0.0453	H = 8.0314
	Mean dose (Gy)	5.37 ± 8.55	5.87 ± 9.06	10.72 ± 6.59	9.38 ± 5.76	0.0001	H = 22.227
	Integral dose (Gy cm^3^)	1.41 ± 2.38	1.50 ± 2.53	1.75 ± 1.61	1.57 ± 1.50	0.9592	F = 0.1009
Left eye	Max dose (Gy)	18.93 ± 10.96	18.53 ± 11.69	10.63 ± 4.51	14.90 ± 7.55	0.0431	H = 8.145
	Mean dose (Gy)	2.62 ± 1.62	2.79 ± 1.89	4.47 ± 1.31	2.66 ± 1.07	0.0001	H = 19.912
	Integral dose (Gy cm^3^)	180.06 ± 115.36	176.72 ± 122.60	98.93 ± 49.88	138.18 ± 77.17	0.0294	F = 3.1583
Right eye	Max dose (Gy)	16.75 ± 12.23	16.74 ± 12.25	12.13 ± 5.10	14.41 ± 7.78	0.0001	F = 1.014
	Mean dose (Gy)	2.91 ± 2.62	2.97 ± 2.64	4.56 ± 1.49	2.89 ± 1.92	0.0031	H = 13.825
	Integral dose (Gy cm^3^)	159.32 ± 123.85	160.65 ± 126.32	112.45 ± 52.94	133.66 ± 82.73	0.3818	F = 1.0354
Brain	Max dose (Gy)	55.17 ± 1.06	54.98 ± 1.05	50.67 ± 1.44	51.22 ± 1.46	<0.0001	F = 71.723
	Mean dose (Gy)	7.29 ± 2.59	7.93 ± 2.83	6.43 ± 2.35	6.15 ± 2.29	0.1084	F = 2.0914
	Integral dose (*10^3^ Gy cm^3^)	64.57 ± 8.22	64.32 ± 7.98	59.37 ± 8.20	59.97 ± 8.00	0.0807	F = 2.3334
Chiasm	Max dose (Gy)	56.41 ± 1.11	55.47 ± 1.05	55.81 ± 0.65	55.91 ± 0.72	0.0065	H = 12.242
	Mean dose (Gy)	55.93 ± 0.95	54.38 ± 2.31	53.96 ± 3.26	54.12 ± 2.64	<0.0001	H = 23.626
	Integral dose (Gy cm^3^)	13.90 ± 16.32	13.60 ± 15.93	13.75 ± 16.15	13.75 ± 16.11	0.9999	F = 0.0013
Brainstem	Max dose (Gy)	50.33 ± 7.19	49.90 ± 7.65	44.18 ± 9.14	47.54 ± 7.34	0.0014	H = 15.5106
	Mean dose (Gy)	21.53 ± 5.17	23.19 ± 4.72	16.92 ± 4.98	20.21 ± 4.76	0.0006	H = 17.163
	Integral dose (*10^3^ Gy cm^3^)	1.25 ± 0.31	1.25 ± 0.33	1.11 ± 0.35	1.18 ± 0.30	0.4882	F = 0.8173

ID analysis demonstrated statistically significant differences among treatment techniques for the optic nerve and eye, indicating that dose deposition varied meaningfully across planning approaches for these structures. In both cases, VMAT showed the lowest ID compared with the other techniques. For all remaining OARs, no statistically significant differences were observed. Overall, although inverse-planned techniques generally showed lower ID trends, significant reductions were limited to selected structures.

## Discussion

This study presents a comprehensive dosimetric comparison of 3DCRT using flattened and FFF beams, IMRT, and VMAT for the treatment of pituitary adenomas. Given the unique anatomical location of pituitary tumors within the sellar and parasellar regions surrounded by radiosensitive structures such as the optic apparatus, brainstem, cochleae, and normal brain, highly conformal radiotherapy techniques are essential to maximize tumor control while minimizing long-term toxicity. The findings of the present analysis demonstrate clear dosimetric advantages of inverse-planned techniques over 3DCRT approaches. Although 3DCRT techniques have historically been relied upon for their simplicity and shorter treatment times, the present findings reinforce the global transition toward IMRT and VMAT for complex anatomical regions where critical structures lie in close proximity to high-dose volumes.

All four techniques achieved clinically acceptable PTV coverage, with ≥95% of the target volume receiving the prescribed dose. However, significant differences were observed in several PTV dose metrics, reflecting the inherent planning characteristics of each modality. The higher minimum PTV dose observed with 3DCRT FFF plans is attributable to the broader high-dose regions and limited modulation capability of forward-planned techniques. In contrast, IMRT demonstrated a lower minimum dose, which is consistent with inverse optimization strategies that prioritize sparing of adjacent OARs when targets abut critical structures [8,9].

Despite these differences, maximum PTV doses did not differ significantly among techniques, indicating effective hotspot control across all modalities. This finding is clinically relevant, as excessive dose heterogeneity within pituitary targets has been associated with increased risk of optic neuropathy and radio-necrosis [10]. The comparable D_98%_ values further suggest that advanced modulation did not compromise target coverage at clinically meaningful dose levels.

CI values were significantly superior for IMRT and VMAT compared with both 3DCRT techniques, underscoring the ability of inverse-planned modalities to better sculpt dose around irregularly shaped intracranial targets. Improved conformity has been consistently reported as a key advantage of IMRT and VMAT in pituitary adenomas and other skull base tumors, where geometric complexity limits the effectiveness of conventional beam arrangements [11].

HI values also favored VMAT and IMRT, with VMAT demonstrating the most uniform dose distribution. Although the absolute differences in HI were small, even modest improvements in dose homogeneity may translate into reduced risk of radiation-induced injury to adjacent optic pathways, particularly in cases where the tumor is in direct contact with the optic chiasm [12].

The GI was lowest for 3DCRT FFF plans, reflecting a steeper dose fall-off immediately outside the target. However, all techniques achieved GI values well below accepted clinical thresholds, indicating acceptable dose gradients overall. Importantly, the improved conformity and organ sparing observed with IMRT and VMAT outweighed the marginal advantage in gradient steepness seen with 3DCRT, supporting the clinical preference for inverse-planned approaches in this setting. These findings parallel those reported in contemporary literature, which consistently highlight the strength of IMRT and VMAT in shaping the high-dose region and minimizing unnecessary spill into surrounding normal tissues [13].

Protection of the optic apparatus remains a key determinant of treatment planning quality in pituitary radiotherapy. In the present study, IMRT achieved the lowest mean doses to selected optic structures, particularly the eye lenses and left optic nerve, indicating effective sparing of small radiosensitive tissues, whereas VMAT consistently produced the lowest maximum doses to the optic nerves and optic chiasm. These findings suggest that inverse-planned techniques provide complementary advantages in optic pathway protection and are consistent with previous reports demonstrating improved sparing of visual structures with advanced planning approaches compared with conventional techniques [14,15].

Eye lens doses were lowest with IMRT and remained well below established cataractogenic thresholds, which is clinically relevant given the potential for cumulative lens exposure to contribute to visual morbidity in long-term survivors of benign pituitary tumors [15]. In contrast, cochlear sparing was most pronounced with 3DCRT FFF, which demonstrated the lowest bilateral cochlear doses, suggesting that beam characteristics may influence dose distribution to laterally positioned structures such as the inner ear [16].

For larger intracranial organs, VMAT achieved the lowest maximum doses to the brain and brainstem, while IMRT demonstrated the lowest mean brain dose, although this difference was not statistically significant. These findings highlight that dose distribution advantages vary among techniques and should be interpreted in the context of anatomical relationships and clinical priorities rather than assuming uniform superiority of any single modality [17-19].

While IMRT provided the most favorable dosimetric profile, it required significantly higher MUs compared with 3DCRT and VMAT. Increased MU usage has been associated with longer beam-on times and higher leakage radiation, although the clinical significance of this effect remains debated [20]. VMAT offered a balance between dosimetric quality and delivery efficiency, achieving conformity comparable to IMRT with substantially fewer MUs and shorter treatment times. This may be particularly advantageous in high-throughput clinical environments or for patients with limited tolerance for prolonged immobilization [21]. Thus, IMRT and VMAT protect OARs better than 3DRT (with or without flattened beams) without compromising the coverage dose to PTV as seen in a previous study with pituitary macroadenomas [13].

True FFF delivery demonstrated lower MU requirements and steeper dose gradients, consistent with previously reported reductions in head scatter and enhanced beam efficiency for unfiltered photon beams [22,23]. In contrast, increased MLC modulation has been associated with greater delivery complexity and elevated peripheral dose, which may explain the slightly improved dose uniformity but higher MU observed with electronically flattened delivery [24,25]. Halcyon-specific studies further support the dominant role of MLC-driven fluence shaping in determining plan quality on this platform [26].

The dosimetric differences observed among techniques have important clinical implications for pituitary adenoma management. Improved sparing of critical structures such as the optic apparatus, cochleae, and brainstem may help reduce the risk of late toxicities, including visual deficits, hearing loss, neurocognitive effects, and brainstem injury. In the present study, inverse-planned techniques demonstrated favorable organ protection overall, with IMRT showing advantages for selected small radiosensitive structures and VMAT providing improved control of maximum doses to larger intracranial organs. These considerations are particularly relevant in benign tumors, where treatment goals extend beyond tumor control to preservation of long-term quality of life.

An important contribution of the present study lies in the characterization of electronically flattened beam delivery on an inherently FFF platform. While previous dosimetric investigations in pituitary adenomas have largely focused on comparisons between conventional 3DCRT, IMRT, and VMAT, the current study specifically evaluated whether dynamic MLC-driven electronic fluence shaping could reproduce clinically relevant forward-planning dose characteristics on the Halcyon system. The observed trade-offs between improved dose homogeneity with electronically flattened delivery and enhanced efficiency and steeper dose gradients with true FFF beams highlight the dosimetric consequences of beam model selection even within forward-planned techniques.

This study, however, is limited by its retrospective and planning-based design on a small sample size. Future investigations should incorporate prospective toxicity assessment, visual and auditory functional testing, and radiobiological modeling such as normal tissue complication probability (NTCP) analysis to compare IMRT and VMAT in treating pituitary adenomas. Emerging techniques, including adaptive radiotherapy and artificial intelligence-driven plan optimization, may further enhance conformity and consistency in pituitary adenoma treatment planning.

Also, the study involved the evaluation of several dosimetric indices, which may increase the risk of false-positive findings due to multiple testing. Formal multiplicity correction methods such as Bonferroni or false discovery rate adjustment were not applied and should be considered in future studies with larger datasets.

The present findings may also have implications for future hypofractionated stereotactic radiotherapy (HSRT) strategies in pituitary adenoma management. Given the favorable conformity, OAR sparing, and delivery efficiency demonstrated by VMAT on the Halcyon platform, advanced inverse-planned approaches may be particularly advantageous for shorter-course treatment regimens. In high-volume clinical environments and resource-constrained settings, hypofractionated schedules could potentially improve machine throughput while simultaneously reducing patient travel, accommodation, and treatment-related logistical burdens. Although the current study was limited to conventionally fractionated plans, further investigation incorporating stereotactic and hypofractionated dose schedules would be clinically valuable.

## Conclusions

This study demonstrates favorable dosimetric characteristics of advanced inverse-planned radiotherapy techniques in the management of pituitary adenomas. Both IMRT and VMAT enable improved dose shaping and organ preservation compared with conventional conformal approaches while maintaining adequate target coverage. Their complementary strengths suggest that technique selection should be guided by anatomical considerations and proximity of critical structures, emphasizing individualized planning rather than reliance on a single modality. Importantly, on the Halcyon platform, true FFF and electronically flattened 3DCRT beam models were not dosimetrically equivalent, with true FFF delivery demonstrating steeper dose gradients and greater efficiency and MLC-based electronic beam flattening producing slightly more homogeneous target dose distributions at the expense of increased fluence modulation, underscoring the clinical relevance of beam model selection even in forward-planned radiotherapy.

## References

[1] Ezzat S, Asa SL, Couldwell WT, Barr CE, Dodge WE, Vance ML, McCutcheon IE (2004). The prevalence of pituitary adenomas: a systematic review. Cancer.

[2] Losa M, Picozzi P, Motta M, Valle M, Franzin A, Mortini P (2011). The role of radiation therapy in the management of non-functioning pituitary adenomas. J Endocrinol Invest.

[3] Otto K (2008). Volumetric modulated arc therapy: IMRT in a single gantry arc. Med Phys.

[4] Gupta T, Chatterjee A (2020). Modern radiation therapy for pituitary adenoma: review of techniques and outcomes. Neurol India.

[5] (2010). Prescribing, recording, and reporting photon-beam intensity-modulated radiation therapy (IMRT): contents. J ICRU.

[6] Paddick I (2000). A simple scoring ratio to index the conformity of radiosurgical treatment plans. Technical note. J Neurosurg.

[7] Paddick I, Lippitz B (2006). A simple dose gradient measurement tool to complement the conformity index. J Neurosurg.

[8] Nutting C, Dearnaley DP, Webb S (2000). Intensity modulated radiation therapy: a clinical review. Br J Radiol.

[9] Webb S (2003). The physical basis of IMRT and inverse planning. Br J Radiol.

[10] Kinaci-Tas B, Alderliesten T, Verbraak FD, Rasch CR (2023). Radiation-induced retinopathy and optic neuropathy after radiation therapy for brain, head, and neck tumors: a systematic review. Cancers (Basel).

[11] Mayo C, Martel MK, Marks LB, Flickinger J, Nam J, Kirkpatrick J (2010). Radiation dose-volume effects of optic nerves and chiasm. Int J Radiat Oncol Biol Phys.

[12] Feuvret L, Noël G, Mazeron JJ, Bey P (2006). Conformity index: a review. Int J Radiat Oncol Biol Phys.

[13] Arauz R, Rodriguez M (2014). RT-02 comparison of 3DCRT, IMRT and VMAT for post surgery radiotherapy for pituitary macroadenomas. Neuro Oncol.

[14] Milano MT, Grimm J, Soltys SG (2021). Single- and multi-fraction stereotactic radiosurgery dose tolerances of the optic pathways. Int J Radiat Oncol Biol Phys.

[15] Ainsbury EA, Dalke C, Hamada N (2021). Radiation-induced lens opacities: epidemiological, clinical and experimental evidence, methodological issues, research gaps and strategy. Environ Int.

[16] Bhandare N, Jackson A, Eisbruch A, Pan CC, Flickinger JC, Antonelli P, Mendenhall WM (2010). Radiation therapy and hearing loss. Int J Radiat Oncol Biol Phys.

[17] Douw L, Klein M, Fagel SS (2009). Cognitive and radiological effects of radiotherapy in patients with low-grade glioma: long-term follow-up. Lancet Neurol.

[18] Minniti G, Traish D, Ashley S, Gonsalves A, Brada M (2005). Risk of second brain tumor after conservative surgery and radiotherapy for pituitary adenoma: update after an additional 10 years. J Clin Endocrinol Metab.

[19] Hall EJ, Wuu CS (2003). Radiation-induced second cancers: the impact of 3D-CRT and IMRT. Int J Radiat Oncol Biol Phys.

[20] Welsh JS, Limmer JP, Howard SP, Diamond D, Harari PM, Tome W (2005). Precautions in the use of intensity-modulated radiation therapy. Technol Cancer Res Treat.

[21] Jiménez-Puertas S, Sánchez-Artuñedo D, Hermida-López M (2018). Assessment of the monitor unit objective tool for VMAT in the Eclipse treatment planning system. Rep Pract Oncol Radiother.

[22] Georg D, Knöös T, McClean B (2011). Current status and future perspective of flattening filter free photon beams. Med Phys.

[23] Vassiliev ON, Titt U, Pönisch F, Kry SF, Mohan R, Gillin MT (2006). Dosimetric properties of photon beams from a flattening filter free clinical accelerator. Phys Med Biol.

[24] Kry SF, Salehpour M, Followill DS, Stovall M, Kuban DA, White RA, Rosen II (2005). Out-of-field photon and neutron dose equivalents from step-and-shoot intensity-modulated radiation therapy. Int J Radiat Oncol Biol Phys.

[25] Ong CL, Dahele M, Slotman BJ, Verbakel WF (2013). Dosimetric impact of the interplay effect during stereotactic lung radiation therapy delivery using flattening filter-free beams and volumetric modulated arc therapy. Int J Radiat Oncol Biol Phys.

[26] Cho S, Cho IJ, Kim YH (2023). Dosimetric analysis of lung stereotactic body radiotherapy using Halcyon linear accelerator. Med Phys.

